# Selections and Abstracts

**Published:** 1908-07

**Authors:** 


### SELECTIONS AND ABSTRACTS

### MEDICAL QUACKERY.

The following letter to the British Medical Journal shows that the American Medical Quack does not limit his exploits to this continent, but brings reproach upon our reputation in other countries.

Only this spring at commencement time an unscrupulous scoundrel was selling diplomas here in Atlanta right under our noses. And yet in our "strenuous inactivity" we meekly submit to quacks and quackery.

Sir,—One of the most common consequences of our ineffective Medical Act is the constant use by unqualified men of so-called American degrees. "M. D. U. S. A.," whatever that may mean, is the "medical degree" usually assumed by the worst characters in this respect. Only a short time ago a person styling himself thus was sentenced to a long term of imprisonment for a grave criminal offence; his career of fraud and crime was for a long time masked and covered by his medical practice, and there is no doubt that, had he not posed as a medical practitioner, he could not with impunity have carried out his many frauds. Lastly I have been investigating the career of a much advertised "Professor" styling himself "M. D., D. Sc. Boston," "the great American doctor," and "eminent American expert." I find that he has spent some portion of his life already in prison for various crimes and misdemeanors, and is at present in custody for other alleged offences against the law. Yet for many years he has succeeded in deluding the public into the belief that he is a reputable American practitioner, and has carried on his frauds under cover of his assumed medical qualifications and many aliases.

Such use of medical titles purporting to hail from America cannot but bring discredit to the country of their assumption.

It behoves the honorable practitioners of America, for whose universities and medical colleges we have a high respect, to do something to put a check upon this reprehensible conduct and to enable the public here to detect the true from the false holders of medical titles.

If the American Medical Association could only induce the Federal Government to issue a State medical register, which could be accepted in our English courts as a register of bona fide medical practitioners of the United States, it would have a good effect and lasting result. At present, in prosecuting these unqualified persons, we are handicapped in not being able to prove the falsity of their pretences in assuming American titles.

It is an interesting factor in unqualified practice that the titles of no other nation are so often utilized by unqualified practitioners as these of the United States. It is also interesting to note that the Medical Acts of the United States are more stringent against unqualified practice than in any other country. Not only has each State its own Medical Act, but each individual practitioner has to take out a license, and the penalties against unqualified practice vary from fines of $25 to $1,000 for each offence; imprisonment, which may be additional to fines, varies from nine days to one year, also doubled for subsequent offences.

A medical practitioner of one State cannot practice in another without taking out a license, which license has to be granted by the State Board of Medicine.

Here in England we have to see daily the use of American medical titles by men who not only have in all probability never been in America, but in addition were they in America and were they to practice medicine would be promptly haled by the District Attorney before a judge and fined and imprisoned. Even our own disregistered men and those from whom English medical qualifications have been removed do not hesitate to add to their names American medical titles. To be removed from the *Medical Register* one day and be "off to Philadelphia in the morning" seems to be the natural sequence in this respect.

I have called the attention of the American Medical Association to the subject of my letter to you, and hope that by the ventilation of this important matter in the British Medical Journal, something may be done to stop this gross scandal, and I hope I shall have your valuable support.—I am, etc.,

A. George Bateman.

Medical Defence Union, 4, Trafalgar Square, London, W. C., May 7th.

---

He Knew.—"Are you in pain, my little man?" asked the kind old gentleman.

"No," answered the boy, "the pain's in me."—Indianapolis Journal.

---

Mr. and Mrs. Hanson Colbert Moseley, of Prosperity, S. C., have issued invitations to the marriage of their daughter, Lula Caroline, to Dr. George Reeves White, of Savannah. The marriage will take place on Tuesday evening, the 30th of June, at 7:30 o'clock, at the home of the bride's parents in Prosperity. Dr. Ralston Lattimore will be Dr. White's best man.

Miss Moseley is a niece of Dr. James Y. Fair.—Sav. Press, June 18.

---

The Kentucky Antituberculosis Association has appropriated $2,500 to buy ten cows for its local sanitarium.
Chicago.

